# Revision of the subterranean genus *Spelaeodiscus* Brusina, 1886 (Gastropoda, Pulmonata, Spelaeodiscidae)

**DOI:** 10.3897/zookeys.769.25258

**Published:** 2018-06-26

**Authors:** Barna Páll-Gergely, Tamás Deli, Zoltán Péter Erőss, Peter L. Reischütz, Alexander Reischütz, Zoltán Fehér

**Affiliations:** 1 Centre for Agricultural Research, Hungarian Academy of Sciences (MTA), Herman Ottó út 15, Budapest, H-1022, Hungary; 2 Móricz Zsigmond u. 2, Gyomaendrőd, H-5500, Hungary; 3 Bem u. 36., Budapest, H-1151, Hungary; 4 Puechhaimg. 52, A-3580, Horn, Austria; 5 Department of Zoology, Hungarian Natural History Museum, Baross u. 13, H-1088, Hungary

**Keywords:** *Aspasita*, Balkans, “Milieu Souterrain Superficiel”, “scratch and flotate”, shell morphology, taxonomy

## Abstract

The Balkan genus *Spelaeodiscus* Brusina, 1886 is revised based on museum collections and newly collected samples from Montenegro and Albania. The following species and subspecies are introduced as new to science: *Spelaeodiscus albanicus edentatus* Páll-Gergely & P. L. Reischütz, **ssp. n.** (southern Montenegro and northern Albania), *Spelaeodiscus densecostatus* Páll-Gergely & A. Reischütz, **sp. n.**, *Spelaeodiscus hunyadii* Páll-Gergely & Deli, **sp. n.**, *Spelaeodiscus latecostatus* Páll-Gergely & Erőss, **sp. n.** (all three from southern Montenegro), *Spelaeodiscus unidentatus acutus* Páll-Gergely & Fehér, **ssp. n.**, and *Spelaeodiscus virpazarioides* Páll-Gergely & Fehér, **sp. n.** (both from northern Albania). For all species and subspecies diagnoses and suggestions for conservation status assessments according to IUCN criteria are provided. An overview is given regarding the habitat preference of *Spelaeodiscus* species, and the “scratch and flotate” method to collect subterranean gastropods.

## Introduction

*Spelaeodiscus* was described by Brusina (1886) as a subgenus of *Patula* Held, 1838. At that time, the only species in this group was P. (S.) hauffeni (Schmidt, 1855). This group (and the only species belonging to it) was known to inhabit Krain (Slovenia) only. The second species of *Spelaeodiscus* was *Spelaeodiscus albanicus* (A. J. Wagner, 1914), which was described from northern Albania, based on shells collected in the debris of the Kir River. *Spelaeodiscus* was first used at the genus level by Pilsbry (1926). In the 1960’s Bole (1961, 1965) and Gittenberger (1969) introduced three additional species as follows: *S. unidentatus* Bole, 1961, *S. obodensis* Bole, 1965 and *S. dejongi* Gittenberger, 1969. The former two were described from present day Montenegro, whereas *S. dejongi* was originally reported from Slovenia. Later it turned out that the Slovenian specimens of *S. dejongi* were probably the result of mislabelling, and that this species is endemic to Montenegro (Gittenberger 1975). Thus, *Spelaeodiscus* is currently known from the Western Balkans (Slovenia, Montenegro, and northern Albania).

*Aspasita* Westerlund, 1889 was established as a “Gruppe” under Helix (Gonostoma), and originally included the three species: *Helix triaria* Rossmässler, 1839 (with its subspecies *tatrica* Hazay, 1883), *Helix trinodis* (Kimakowicz, 1884), and *H. triadis* (Kimakowicz, 1884). Gittenberger (1969) recognized a single species, Spelaeodiscus (Aspasita) triaria, and treated the other taxa as its subspecies (*trinodis*, *triadis*, *tatricus*). Another *Aspasita* species, *A. bulgarica* Subai & Dedov, 2008, has been described recently from Bulgaria. Currently, *Aspasita* is known from the Northern Carpathians (Tatra Mts in Slovakia, Bükk Mts in Hungary), the Apuseni Mts and the Southern Carpathians (Romania), and the Stara Planina Mts (Bulgaria and northeastern Serbia).

*Aspasita* and *Spelaeodiscus* have been distinguished by Schileyko (1998) and Subai and Dedov (2008) on the genus, by Gittenberger (1969, 1975) on the subgenus level. However, Welter-Schultes (2012) treated them as a single genus. In this paper we treat the two genera separately.

The conchologically similar genus *Virpazaria* Gittenberger, 1969, which is also an endemic of the West Balkans, is distinguished from *Spelaeodiscus* and *Aspasita* on the basis of the continuous peristome and the crescent-shaped aperture (Gittenberger 1969, 1975, Reischütz and Reischütz 2009).

So far, *Spelaeodiscus* species have mainly been reported from caves, and thus, they belong to the rarest genera in mollusc collections. Intensive field surveys in Montenegro and Albania, and using special collecting methods (sieving and flotating the granular rocky substrate collected from rock crevices) significantly increased the number of known populations and the amount of the available shell material of *Spelaeodiscus*. In the present revision we present the outcome of the examination of all available historical and newly collected material.

## Materials and methods

The new samples were collected between 2010 and 2017 during 13 collecting trips. Sampling was done scratching out fine granulate material from the superficial fissures of rocks applying long and narrow hand rakes and separated the shells either by sieving (“scratch and sieve” method) or by flotating (“scratch and flotate” method).

**Figure 1.**
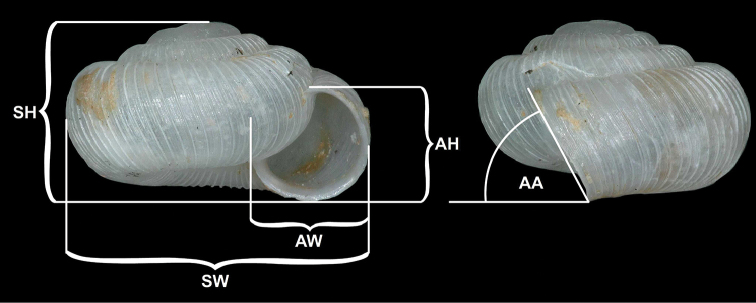
The following variables were measured: angle of aperture (AA); aperture height (AH); aperture width (AW); shell height (SH); shell width (SW).

Shell whorls (± 0.25) were counted according to Kerney and Cameron (1979: 13). Most shells were measured in mm to one significant digit using Zeiss Stemi 305 microscope with Zeiss Labscope software. The measured parameters are compiled on Figure 1. Ribs on the body whorl were counted using photographs of 3–5 shells per population. Differences in size are indicated in the diagnoses using the following terms: small (1.9–2.5 mm), medium sized (2.6–3.5 mm), large (3.6–4.3 mm).

### Abbreviations used

**HNHM** Hungarian Natural History Museum (Budapest, Hungary)

**DT** Collection Tamás Deli (Gyomaendrőd, Hungary)

**EZP** Collection Zoltán Péter Erőss (Budapest, Hungary)

**HA** Collection András Hunyadi (Budapest, Hungary)

**JG** Collection Jozef Grego (Banská Bystrica, Slovakia)

**MZBI** Jovan Hadži Institute of Biology, Research Centre of the Slovenian Academy of Sciences and Arts (Ljubljana, Slovenia)

**NHMW** Naturhistorisches Museum Wien (Vienna, Austria)

**NMBE** Natural History Museum of Bern (Bern, Switzerland)

**PGB** Collection Barna Páll-Gergely (Mosonmagyaróvár, Hungary)

**REI** Collection Reischütz (Horn, Austria)

**SMF** Senckenberg Forschungsinstitut und Naturmuseum (Frankfurt am Main, Germany)

## Systematics

### Family Spelaeodiscidae

Taxon classificationAnimaliaStylommatophoraSpelaeodiscidae

Steenberg, 1925

#### Remarks.

Schileyko (1998) classified *Spelaeodiscus* into the family Spelaeodiscidae Steenberg, 1925, which has independently been introduced by Hudec (1970) as well. This taxon was also recognized as a separate family of the infraorder Orthurethra (and its only superfamily, Pupilloidea) by Bouchet et al. (2017). Although the genital anatomy of some species belonging to this family is known, its systematic position within Orthurethra is uncertain, because no molecular phylogenetic information is known (Harl et al. 2017).

### Genus Spelaeodiscus

Taxon classificationAnimaliaStylommatophoraSpelaeodiscidae

Brusina, 1886

Patula (Spelaeodiscus) Brusina, 1886: 37.

#### Type species.

*Helix Hauffeni* Schmidt, 1855.

#### Distribution.

**Figure 2.**
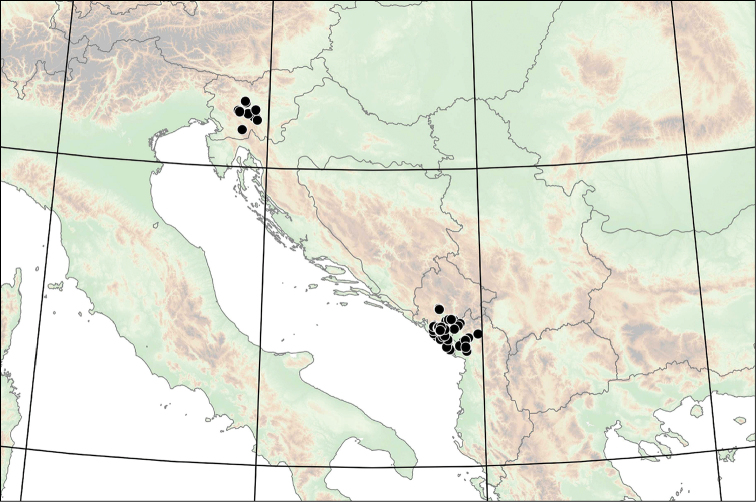
Distribution of the genus *Spelaeodiscus* Brusina, 1886.

The genus *Spelaeodiscus* has a disjunct distribution. One species (*S. hauffeni*) is only known from Slovenia, whereas the rest of the genus is distributed in the vicinity of the Skadar Lake Basin (also known as Shkodër Lake or Skutari Lake) in Montenegro and northern Albania (Figure 2).

#### Included taxa.

*Spelaeodiscus albanicus albanicus* (A. J. Wagner, 1914), *S. albanicus edentatus* Páll-Gergely & P. L. Reischütz, ssp. n., *S. dejongi* Gittenberger, 1969, *S. densecostatus* Páll-Gergely & A. Reischütz, sp. n., *S. hauffeni* (Schmidt, 1855) *S. hunyadii* Páll-Gergely & Deli, sp. n., *S. latecostatus* Páll-Gergely & Erőss, sp. n., *S. obodensis* Bole, 1965. *S. unidentatus unidentatus* Bole, 1961, *S. unidentatus acutus* Páll-Gergely & Fehér, ssp. n., *S. virpazarioides* Páll-Gergely & Fehér, sp. n. For key traits see Table 1.

**Table 1.** Number of ribs on the body whorl, shell size, and key traits of *Spelaeodiscus* species.

(Sub)species	No. of ribs	Shell diameter (in mm)	Key traits
*albanicusalbanicus*	43–93	3.6–4.3	matte protoconch, weak palatal and two weak basal teeth
*albanicus edentatus* ssp. n.	35–54	3.6–4.2	widely-spaced ribs, glossy protoconch glossy, toothless aperture
*dejongi*	57–112	1.9–3.4	dense, low ribs, smooth protoconch, toothless aperture
*densecostatus* sp. n.	116	3.7	very low and dense ribs, toothless aperture
*hauffeni*	41–52	2.8–3.5	widely spaced, strong ribs, rounded, toothless aperture, finely granular protoconch
*latecostatus* sp. n.	42	2.2	strong, very widely spaced ribs, glossy protoconch, toothless aperture
cf. latecostatus sp. n. (2017/005)	47–54	1.9	strong, widely spaced ribs, glossy protoconch, toothless aperture
*hunyadii* sp. n.	42–48	2.1–2.2	widely spaced, strong ribs, glossy protoconch, strongly oblique, toothless aperture
*obodensis*	43–76	2.6–3.0	elevated spire, roughly sculptured protoconch, strong ribs, toothless aperture
*unidentatusunidentatus*	74–118	2.4–3.2	low basal tooth; palatal part of peristome with strong incision
*unidentatus acutus* ssp. n.	64–91	2.9–3.5	pointed basal tooth; palatal part of peristome with shallow incision
*virpazarioides* sp. n.	40–70	3.3–3.6	spiral sculpture, thickened callus

#### Delimitation of this genus.

The reproductive anatomy of *Spelaeodiscus* and *Aspasita* is characterized by a short penial caecum, a well-developed penial appendix, sometimes an epiphallic caecum, and a bursa copulatrix without a diverticulum. The retractor muscle is divided into two bounds, one inserting on the penial appendix, whereas the other at the base of the penial caecum. Examining the anatomical descriptions and drawings of *Spelaeodiscus* (Bole 1965) and *Aspasita* (Hudec 1965, Gittenberger 1975, Schileyko 1998, Subai and Dedov 2008), we were unable to find characters that would constantly differ between the two groups. For example, the penial caecum was long and slender in *A. tatrica* (see Hudec 1965) and *S. hauffeni* (see Bole 1965), but was short and conical in *A. triaria* (see Subai and Dedov 2008) and *S. unidentatus* (see Bole 1965). Also, the shape of the bursa and the position of the starting point of the penial appendix was greatly variable across genera. Clear epiphallic caecum was only found in *S. hauffeni*, but some thickening was visible in *S. unidentatus* and *A. triaria*.

As for shell characters, *Spelaeodiscus* is characterized by a mostly colourless shell that is smaller than 4.3 mm (majority of species are even smaller than 3.5 mm), the spire is relatively low (height of body whorl at least two third of the height of the entire shell), the body whorl is evenly rounded, the edge of the parietal callus is straight, and the peristome is only slightly expanded. In contrast, *Aspasita* shells are brownish, larger than 4.3 mm, they have higher spire (height of body whorl is approximately half of the height of the entire shell), the shell is shape reverse trapezoid from standard apertural view, the callus is heart-shaped, and the basal part of the peristome is strongly expanded.

The habitat was the only “trait” mentioned by Gittenberger (1969) as difference between the two groups. Namely, *Spelaeodiscus* is subterranean, whereas *Aspasita* can be found on rock surfaces and among leaf litter at the base of limestone rocks. In the lack of sound molecular data it is difficult to infer their relationship, but based only on ecological, conchological, and biogeographical differences it seems reasonable to keep *Aspasita* and *Spelaeodiscus* as distinct genera.

### Spelaeodiscus albanicus

Taxon classificationAnimaliaStylommatophoraSpelaeodiscidae

(A. J. Wagner, 1914)

#### Diagnosis.

A large species with usually widely spaced, strong ribs, and no or weak apertural teeth.

#### Differential diagnosis.

The most similar species in terms of shell size and shape is *Spelaeodiscus densecostatus* sp. n., for differences see under that species. *Spelaeodiscus unidentatus* is usually smaller, usually possesses denser ribs, and has stronger teeth and narrower aperture.

#### Conservation status.

Reischütz and Fehér (2017) assessed this species as Least Concern (LC). They claimed that there are at least three known locations and is likely that further field work reveals a larger range and more locations. Although that assessment was based partly on incorrect distribution records (Peuta Cave population is currently treated as *S. unidentatus*, whereas Raps-Starjë population as *S. virpazarioides* sp. n.), together with our new distribution records there are more than five locations. As we have no reason to suppose that the habitat quality, habitat extent, or population are deteriorating or extremely fluctuating, Least Concern (LC) seems to be a correct assessment.

### Spelaeodiscus albanicus albanicus

Taxon classificationAnimaliaStylommatophoraSpelaeodiscidae

(A. J. Wagner, 1914)

Figure 3

Aspasita albanica A. J. Wagner, 1914 in Sturany & Wagner 1914: 67, plate 2, figs 10a–c.
Spelaeodiscus albanicus — Bole 1965: 354, Plate 76, fig. B.
Spelaeodiscus (Spelaeodiscus) albanicus — Gittenberger 1969: 294, fig. 2.
Spelæodiscus albanicus — Pilsbry 1926: 184, plate 22, figs 12–14.
Spelaeodiscus albanicus — Welter-Schultes 2012: 213 (partim: locality data from Montenegro are of unknown origin).
Spelaeodiscus albanicus — Reischütz et al. 2013: 62, fig. 3.

#### Type material.

**Figure 3.**
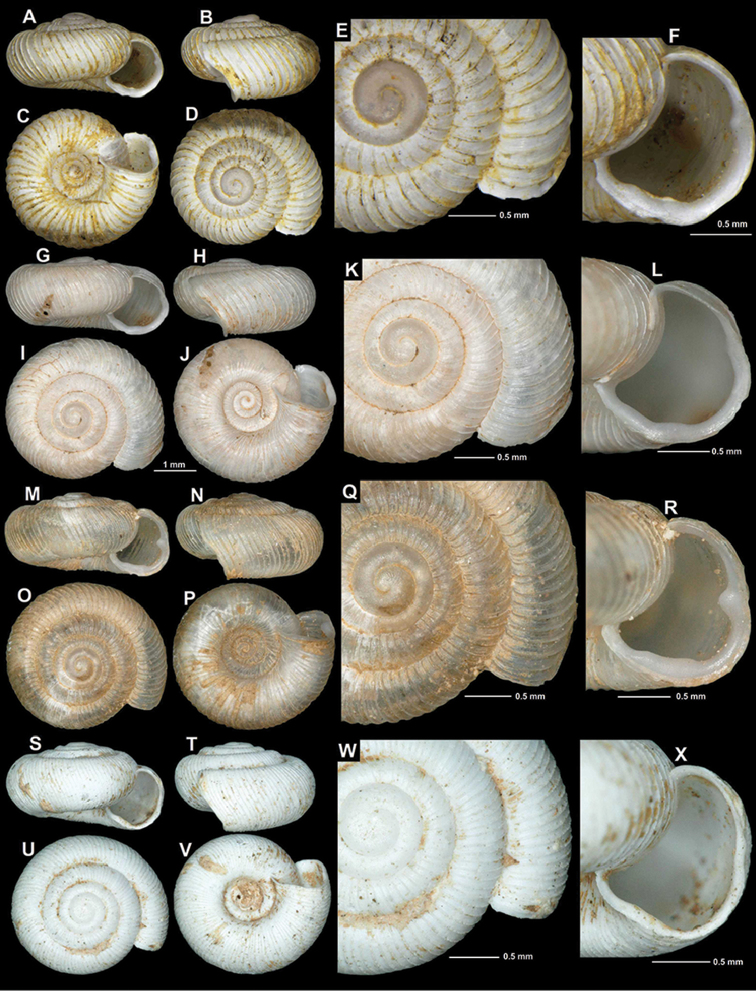
Shells of *Spelaeodiscus albanicus albanicus* (A. J. Wagner, 1914). **A–F** lectotype (NHMW 43385) **G–L** Albania, Drisht, right bank of Kir river opposite to the fortress hill (HNHM 103195) **M–R** Albania, Shkodër district, 1 km NE of Ura e Shtrenjtë (HNHM 103196) **S–X** Albania, rocks southwest of Zusi (NHMW 112358).

Kiri-Brücke nächst Mesi b. Skutari, Albanien (im Genist), leg. Sturany, 27.04.1905, NHMW 43385 (lectotype, hereby selected, SW: 3.7 mm, SH: 2 mm, Fig. 3A–F); Drinasca-Ufer b. Skutari (angeschwemmt), leg. Sturany, 03.05.1905, NHMW 112351 (1 corroded, juvenile paralectotype).

#### Other material.

Vrelo Pronisicut (or Pronifkut), coll. Edlauer ex coll. Kuščer, NHMW 48236/1 shell (“photo”); Pronisicut, coll. Edlauer ex coll. Kuščer, NHMW48336/2 shells; Albania, Shkodër district, Drisht, right bank of Kir river opposite to the fortress hill, 90 m a.s.l. (roadside limestone rocks), 42°7.824'N, 19°36.540'E, (site code: 2016/32), leg. Z.P. Erőss, Z. Fehér, J. Grego & M. Szekeres, 28.06.2016, HNHM 103195/1 (photographed shell, Fig. 3G–L), NHMW 112356/1 adult + 5 juvenile shells; Albania, Shkodër district, 1 km NE of Ura e Shtrenjtë, 160 m a.s.l., (roadside limestone rocks), 42°9.180'N, 19°40.000'E (site code: 2016/33), leg. Z.P. Erőss, Z. Fehér, J. Grego & M. Szekeres, 28.06.2016, HNHM 103196/1 (photographed shell, Fig. 3M–R), NHMW 112357/9 juvenile/broken shells; Albania, Shkodër district, 4 km SW of Prekal, 170 m a.s.l., (roadside limestone rocks), 42°9.936'N, 19°41.334'E (site code: 2016/35), leg. Z.P. Erőss, Z. Fehér, J. Grego & M. Szekeres, 28.06.2016, JG/1 adult + 1 juvenile shell; Albania, Ura e Mesit, debris of the Kir river, 50 m a.s.l., 42°6.870'N, 19°34.498'E, leg. A. Reischütz, N. Reischütz & P. L. Reischütz, Apr. 2012, REI/1; Albania, rocks southwest of Zusi, southwest of Skoder, 13 m a.s.l., 42°2.316'N, 19°28.866'E, leg. A. Reischütz, N. Reischütz & P. L. Reischütz, May 2015, NHMW 112358/1 (photographed shell, Fig. 3S–X), REI/5; Albania, Felsspalten above Drisht at Mes, 115 m a.s.l., 42°7.735'N, 19°36.823'E, leg. A. Reischütz, N. Reischütz & P. L. Reischütz, Jul. 2010, REI/2 juvenile shells; Albania, ruins 3 km above Drisht at Mes, 195 m a.s.l., 42°7.468'N, 19°36.846'E, leg. A. Reischütz, N. Reischütz & P. L. Reischütz, Jul. 2010, REI/3; Albania, Periferi Shkodër, ca. 18 km upstream from dam at Koman, a left side-valley of Liqeni i Komanit, 170 m a.s.l., limestone rocks, debris, 42°13.613'N, 19°54.300'E, leg. Z.P. Erőss, Z. Fehér, A. Hunyadi & D. Murányi, 15 Apr. 2006, HNHM 102244/1.

#### Diagnosis.

Protoconch matte; aperture with a rather weak palatal and two weak basal teeth.

#### Description.

Shell rarely flat, usually spire somewhat elevated; protoconch consists of 1.5–1.75 whorls, very finely granulated, rather matte, not glossy; teleoconch with strong, equidistant ribs that are supported by fine periostracal filaments in fresh shells; rib density variable (43–93 ribs on body whorl), usually widely spaced; between main ribs some fine wrinkles discernible; entire shell with 4.25–4.5 whorls; aperture semilunar or due to the straight basal part triangular; peristome expanded and slightly reflected, especially on the palatal, basal and umbilical areas; palatal tooth of variable strength, usually weak, although present in all adult shells, palatal region of peristome without outer incision; basal portion of peristome usually straight, slightly thickened, with two low denticles that are visible in all adult shells; umbilicus funnel-shaped, wide (although width depends on spire height).

**Measurements.** SW: 3.6–4.3 mm (median = 4.0 mm), SH: 1.9–2.2 mm (median = 2.0 mm), AW: 1.3–1.7 mm (median = 1.5 mm), AH: 1.3–1.6 mm (median = 1.5 mm), (n = 6; largest and smallest specimens of multiple populations measured).

#### Differential diagnosis.

See under *S. albanicus edentatus* ssp. n. Densely ribbed specimens of this subspecies might resemble large specimens of *S. unidentatus*. The latter species, however, usually has stronger teeth, and narrower aperture.

#### Variation among specimens.

Some variability was found between populations in terms of rib density, spire height, and strength of apertural teeth.

#### Distribution.

**Figure 4.**
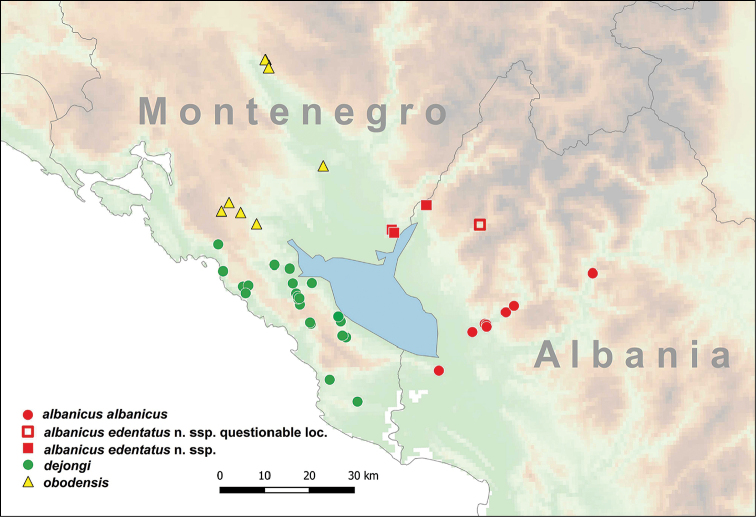
Distribution of *Spelaeodiscus* Brusina, 1886 species.

This subspecies is distributed in northwestern Albania. Most of the known distribution records are from the Kir River Valley. One shell found in the fluvial debris of the Drin River above the Koman Dam extends the range farther eastwards (Figure 4). Welter-Schultes (2012) erroneously reports this taxon from Montenegro.

#### Conservation status.

See under *Spelaeodiscus albanicus*.

### Spelaeodiscus albanicus edentatus

Taxon classificationAnimaliaStylommatophoraSpelaeodiscidae

Páll-Gergely & P. L. Reischütz ssp. n.

http://zoobank.org/D00AA0E9-7121-451A-91C0-25655AAAED3C

Figure 5A–L

Spelaeodiscus sp. (aff. obodensis) — Reischütz et al. 2013: 62.

#### Type material.

**Figure 5.**
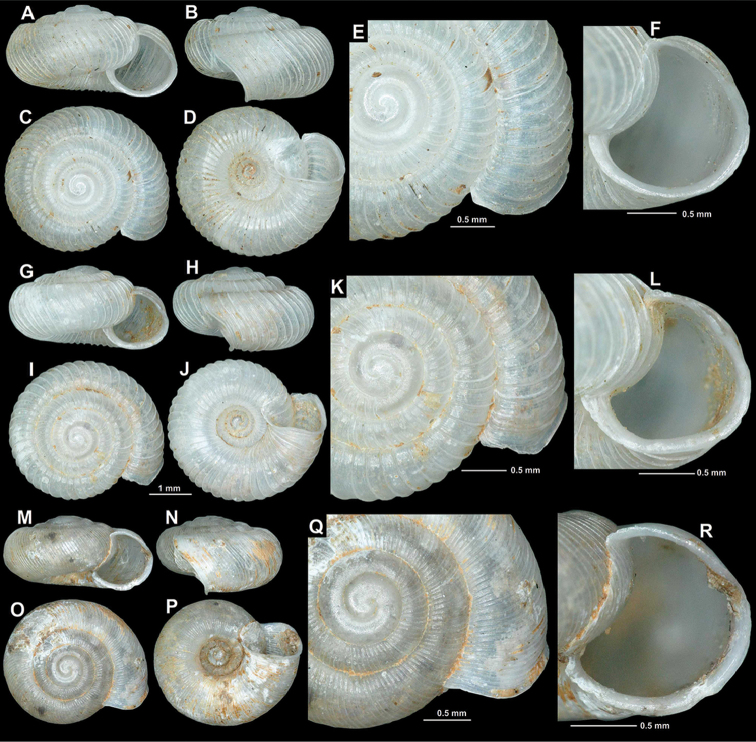
Shells of *Spelaeodiscus* Brusina, 1886 species. **A–F** *Spelaeodiscus albanicus edentatus* ssp. n., holotype (NHMW 112360) **G–L** *Spelaeodiscus albanicus edentatus* ssp. n., Montenegro, rocks across the road at Vitoja, Skadarsko Jezero (NHMW 112361) **M–R** *Spelaeodiscus densecostatus* sp. n., holotype (NHMW 112364).

Albania, rocks along the road Hani i Hotit to Vermosh, 7.2–7.8 km north of the junction, 370 m a.s.l., 42°22.451'N, 19°27.507'E, leg. A. Reischütz, N. Reischütz & P. L. Reischütz, Apr. 2014, NHMW 112360/1 (holotype, SW: 4.2 mm, SH: 2.2 mm, Fig. 5A–F), REI/4 paratypes; rocks above Hani i Hotit, leg. A. Reischütz, N. Reischütz & P. L. Reischütz, Apr. 2012, REI/1 paratypes; Montenegro, Podgorica Municipality, Izvor Vitoja S 1 km, near Shkodra Lake, 30 m, limestone rocks, 42°19.176'N, 19°22.158'E (site code: 2015/105), leg. Z.P. Erőss, Z. Fehér, J. Grego, 05 Jul. 2015, HNHM 103197/4 paratypes, JG/4 paratypes, NHMW 112359/4 paratypes; Montenegro, rocks across the road at Vitoja, Skadarsko Jezero, 16 m a.s.l., 42°19.554'N, 19°21.776'E, leg. A. Reischütz, N. Reischütz & P. L. Reischütz, Apr. 2012, NHMW 112361/1 (photographed paratype, Fig. 5G–L), REI/4 paratypes.

#### Additional material.

Albania, Malesia district, Xhajë NE 0.5 km, 650 m a.s.l., 42°19.838'N, 19°36.341'E (site code: 2015/103), rocks, leg. Z.P. Erőss, Z. Fehér & J. Grego, 04.07.2015, HNHM 103493/5 juvenile shells (not paratypes); NHMW 110430/MN/0985/5 juvenile shells (not paratypes), JG/6 juvenile shells (not paratypes).

#### Type locality.

Albania, rocks along the road Hani i Hotit to Vermosh, 7.2–7.8 km north of the junction, 370 m a.s.l., 42°22.451'N, 19°27.507'E.

#### Diagnosis.

Protoconch glossy; aperture without teeth.

**Measurements.** SW: 3.6–4.2 mm (median = 4.0 mm), SH: 1.9–2.2 mm (median = 2.1 mm), AW: 1.5–1.7 mm (median = 1.6 mm), AH: 1.3–1.6 mm (median = 1.4 mm), (n = 6; largest and smallest specimens of multiple populations measured).

#### Differential diagnosis.

This new subspecies differs from the nominotypical subspecies in the following traits: aperture relatively larger, and its basal area not straight; apertural barriers (teeth) absent; protoconch smooth, glossy; ribs somewhat less dense (rib density on body whorl: 35–54).

#### Variation among specimens.

This subspecies shows some variability in terms of shell size and rib density.

#### Etymology.

This new subspecies is named after its toothless aperture, which distinguishes it from the nominotypical subspecies.

#### Distribution.

This taxon is found in the northeastern part of the Lake Shkodër Basin (Figure 4).

#### Conservation status.

See under *Spelaeodiscus albanicus*.

### Spelaeodiscus dejongi

Taxon classificationAnimaliaStylommatophoraSpelaeodiscidae

Gittenberger, 1969

Figure 6

Spelaeodiscus (Spelaeodiscus) dejongi Gittenberger, 1969: 295–296, fig. 3.
Spelaeodiscus dejongi — Welter-Schultes 2012: 213.

#### Type material.

**Figure 6.**
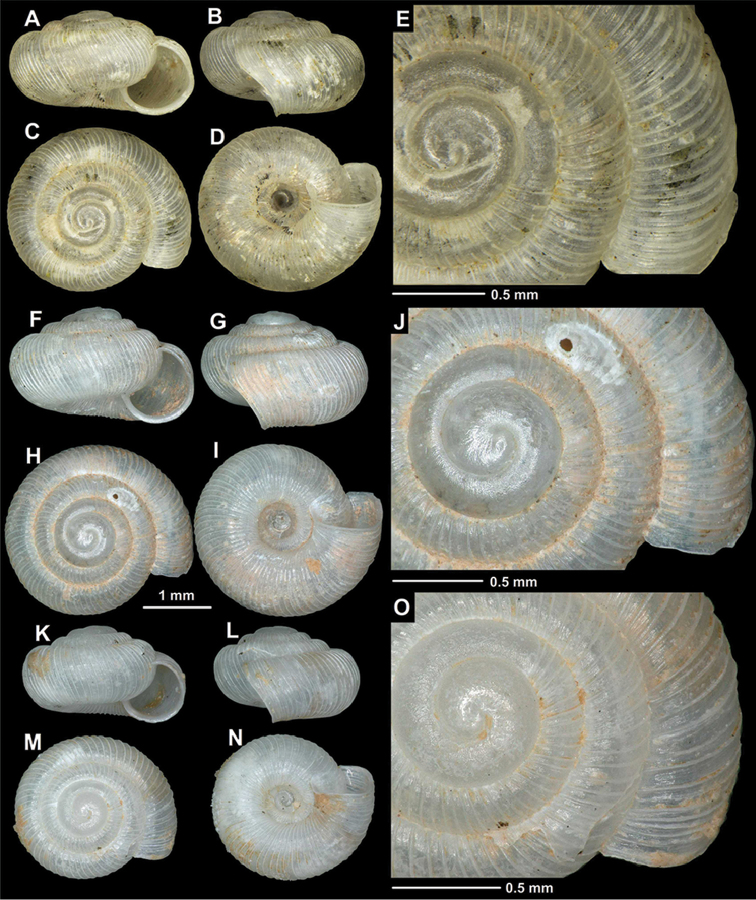
Shells of *Spelaeodiscus dejongi* Gittenberger, 1969. **A–E** holotype (NHMW 42517) **F–J** Montenegro, Gradiste Monastery (NHMW 112362) **K–O** Montenegro, 1.9 km (in a straight line) S of Virpazar (HNHM 103200).

Jama Nadjama bei Gnezdu (Izitovice), Krain, leg. Kuščer, coll. Edlauer, NHMW 49517a (holotype, SW: 2.8 mm, SH: 1.6 mm, Fig. 6A–E); Same data, NHMW 49517b/17 paratypes. See remarks concerning the type locality.

#### Other material.

Vetajama bei Sokol, coll. Edlauer, NHMW 48071/2, (det. Gittenberger, 1974); Radetina pećina, Itijino brdo, 1300 m, leg. Dabović, NHMW 48312/11 (large shells, similar to the type); Pećina Marka Vuksanovića, 1400 m, leg. Dabović, coll. Edlauer ex coll. Kuščer 666/10, NHMW 49321/1 (det. Gittenberger, 1974); Montenegro, S of Virpazar, 1 km (in a straight line) ESE of Limljani, near the small road, 323 m a.s.l., 42°11.414'N, 19°06.277'E (site code: 20171019B), leg. T. Deli, Z.P. Erőss, A. Hunyadi & B. Páll-Gergely, 19.10.2017, DT/2, HA/1, HNHM 103198/2, PGB/1; Montenegro, S of Virpazar, 0.8 km (in a straight line) NE of Limljani, near the small road, 350 m a.s.l., 42°12.068'N, 19°05.969'E (site code: 20171019C), leg. T. Deli, Z.P. Erőss, A. Hunyadi & B. Páll-Gergely, 19.10.2017, DT/2, HA/2. HNHM 103199/2, PGB/1; Montenegro, 1.9 km (in a straight line) S of Virpazar, near the road, 160 m a.s.l., 42°13.312'N, 19°05.440'E (site code: 20171019E), leg. T. Deli, Z.P. Erőss, A. Hunyadi & B. Páll-Gergely, 19.10.2017, DT/24, EZP/24, HA/24, HNHM 103200/24 + 1 photographed shell (Fig. 6K–O), PGB/24; Montenegro, SE of Virpazar, 4.3 km (in a straight line) SSE of Ðuravci, near Besa/Bes near Krone i Besit, 330 m a.s.l., 42°08.548'N, 19°13.165'E (site code: 20171019G), leg. T. Deli, Z.P. Erőss, A. Hunyadi & B. Páll-Gergely, 19.10.2017, DT/60–70, EZP/60–70, HA/60–70, HNHM 103201/28, PGB/60–70; Montenegro, SE of Virpazar, 7.3 km S(SE) of Ðuravci, 1.6 km (in a straight line) NNW of Tejani, 455 m a.s.l., 42°06.803'N, 19°13.384'E (site code: 20171019I), leg. T. Deli, Z.P. Erőss, A. Hunyadi & B. Páll-Gergely, 19.10.2017, HNHM 103202/2; Montenegro, NNW of Virpazar, road between Rijeka Crnojevića and Virpazar, at the junction to Dupilo, 160 m a.s.l., 42°15.085'N, 19°04.987'E (site code: 20171020A), leg. T. Deli, Z.P. Erőss, A. Hunyadi & B. Páll-Gergely, 20.10.2017, HNHM 103203/1; Montenegro, S of Virpazar, 1.1 km (in a straight line) E of Limljani, one of the tunnels on the old road, 400 m a.s.l., 42°11.637'N, 19°06.309'E (site code: 20171021A), leg. T. Deli, Z.P. Erőss, A. Hunyadi & B. Páll-Gergely, 21.10.2017, DT/ca. 20, EZP/ca. 20, HA/ca. 20, HNHM 103204/6, PGB/ca. 20; Montenegro, S of Virpazar, 0.8 km (in a straight line) E of Limljani, above the village, 400 m a.s.l., 42°11.698'N, 19°06.217'E (site code: 20171021B), leg. T. Deli, Z.P. Erőss, A. Hunyadi & B. Páll-Gergely, 21.10.2017, DT/ca. 40, EZP/ca. 40, HA/ca. 40, HNHM 103205/8, PGB/ca. 40; Montenegro, NE of Bar, 1.2 km (in a straight line) NW of Tudjemili, 400 m a.s.l., 42°08.489'N, 19°08.144'E (site code: 20171021F), leg. T. Deli, Z.P. Erőss, A. Hunyadi & B. Páll-Gergely, 21.10.2017, DT/ca. 40, EZP/ca. 40, HA/ca. 40, HNHM 103206/10, PGB/ca. 40; Montenegro, 1.6 km (in a straight line) NE Petrovac, 0.3 km S of Novoselje, 470 m a.s.l., 42°13.032'N, 18°57.336'E (site code: 20171021G), leg. T. Deli, Z.P. Erőss, A. Hunyadi & B. Páll-Gergely, 21.10.2017, EZP/3; Montenegro, 2.8 km (in a straight line) NE Petrovac, 1.2 km E of Novoselje, 630 m a.s.l., 42°13.145'N, 18°58.245'E (site code: 20171021H), leg. T. Deli, Z.P. Erőss, A. Hunyadi & B. Páll-Gergely, 21.10.2017, DT/3, EZP/2, HA/4, HNHM 103207/1, PGB/2; Montenegro, Gradiste Monastery, 80 m a.s.l., 42°12.212'N, 18°57.772'E, leg. A. Reischütz, N. Reischütz & P. L. Reischütz, Jul. 2010, NHMW 112362/1 (photographed shell, Fig. 6F–J); REI/5; Montenegro, above Sv. Stefan south of Budva, 110 m a.s.l., 42°14.926'N, 18°54.131'E, leg. A. Reischütz, N. Reischütz & P. L. Reischütz, Mar. 2011, REI/4; Montenegro, Tuđemili, 17 km towards Virpazar, Rumija Mts, 480 m a.s.l., 42°10.974'N, 19°6.546'E, leg. P. Subai, 15 Apr. 2009, NMBE 542070/56; Montenegro, Tuđemili, 1 km towards Virpazar, Rumija Mts, 395 m a.s.l., 42°8.238'N, 19°8.388'E, leg. P. Subai, 25 Sep. 2005, NMBE 542069/1; Montenegro, Tuđemili, 1 km towards Virpazar, Rumija Mts, 395 m a.s.l., 42°8.238'N, 19°8.388'E, leg. P. Subai, 25 Sep. 2005, NMBE 542068/3; Montenegro, Ðuravci, 10 Km E, southern side of Mount Kronistar, 500 m a.s.l., 42°6.594'N, 19°13.908'E, leg. P. Subai, 16 Sep. 2006, NMBE 542067/1; Montenegro, Zoganje N 5 km, on the Ulcinj-Shkodër road, 80 m a.s.l., 41°58.762'N, 19°15.547'E (site code: 2015/32), leg. T. Deli, Z.P. Erőss & Z. Fehér, 27 May 2016, DT/ca. 50, HNHM 103208/22, NHMW 112363/ca. 50; Montenegro, Rumija Mts, Virpazar S 9 km (Virpazar–Bar road, between Boljevići and Tuđemili), old tunnel, 440 m, 42°11.474'N, 19°06.489'E (site code: 2008/182), leg. Z. Fehér, J. Kontschán & D. Murányi, 14.10.2008, HNHM 103209/23; Montenegro, scree (talus) ca. 5 km east of Dobra Voda in direction of Vladimir, 220 m a.s.l., 42°1.508'N, 19°11.160'E, leg. A. Reischütz, N. Reischütz & P. L. Reischütz, Jul. 2010, REI/6; Montenegro, Donji Murići, above the village, at the junction of a minor road to Besa, 200 m a.s.l., 42°9.233'N, 19°12.930'E (site code 2017/007), leg. Z.P. Erőss & Z. Fehér 16.07.2017, HNHM 103492/1 (juvenile shell, identification uncertain).

#### Diagnosis.

A small to medium sized species with dense, low ribs, smooth protoconch, and toothless aperture.

#### Description.

Shell nearly flat, but spire somewhat always elevated; protoconch consists of 1.25–1.75 whorls, rather glossy; teleoconch with fine, equidistant, dense ribs; rib density variable (57–112 ribs on body whorl); between main ribs some fine wrinkles discernible; entire shell with 3.25–3.75 whorls; aperture semilunar; peristome slightly thickened and expanded; aperture toothless; umbilicus regular funnel-shaped, relatively narrow (width depends on spire height).

**Measurements.** SW: 1.9–3.4 mm (median = 2.3 mm), SH: 1.1–1.7 mm (median = 1.3 mm), AW: 0.7–1.3 mm (median = 0.9 mm), AH = 0.8–1.3 mm (median = 0.9 mm), AA = 56–68°(n = 15).

#### Differential diagnosis.

See under *S. obodensis* and *S. hunyadii* sp. n.

#### Variation among specimens.

This is a widely distributed species with numerous known populations, most of them with unique character states of spire height, shell size, and rib density.

#### Distribution.

This species is distributed in the Rumija Mountain between the Shkodër Lake Basin and the Adriatic Sea. Northwards the range extends to the Cetinje area. According to the original labels, type material is of Slovenian origin, however, this species was never again found in Slovenia. It can be reasonably supposed that it is due to mislabelling and the ‘type locality’ is not the site where the type material actually came from (Gittenberger 1975). Welter-Schultes (2012) reports the species only from Slovenia, which is based on the originally incorrect type locality.

#### Conservation status.

Assessed as Least Concern (LC) by Reischütz (2017a), because it is not an extremely rare species and there is no reason to suppose that the habitat quality, habitat extent or population are deteriorating or extremely fluctuating. Now, the number of known locations is more than 20, which confirms the LC status.

### Spelaeodiscus densecostatus

Taxon classificationAnimaliaStylommatophoraSpelaeodiscidae

Páll-Gergely & A. Reischütz sp. n.

http://zoobank.org/DDB4A406-1BF8-4062-A6F4-FB723C513FF8

Figure 5M–R

#### Type material.

Montenegro, Hotel/Restaurant Izvor north of Sutomore, no GPS available, leg. A. Reischütz, N. Reischütz & P. L. Reischütz, May 2015, NHMW 112364 (holotype: SW = 3.7 mm, SH = 1.8 mm, Figure. 5M–R), REI/1 juvenile/broken paratype.

#### Type locality.

Montenegro, Hotel/Restaurant Izvor north of Sutomore.

#### Diagnosis.

A large species with very low and dense ribs; aperture toothless.

#### Description.

Spire slightly elevated; protoconch consists of slightly more than 1.5 whorls, rather glossy; teleoconch with very fine, low, equidistant riblets (approx. 112 on the body whorl); between main ribs some fine wrinkles discernible; entire shell with 3.75 whorls; aperture semilunar, peristome; peristome expanded and slightly reflected on the basal and umbilical areas; aperture toothless; umbilicus regular funnel-shaped, relatively wide.

**Measurements.** SW = 3.7 mm, SH = 1.8 mm, AW = 1.5 mm, AH = 1.4 mm (holotype).

#### Differential diagnosis.

*Spelaeodiscus densecostatus* sp. n. differs from *S. albanicus* by the smaller shell and the much denser and lower ribs on the teleoconch. Furthermore, *S. albanicus albanicus* has two basal and a palatal tooth in the aperture.

#### Variation among specimens.

The only known adult shell is the holotype. Therefore, the morphological diversity within population is unknown.

#### Etymology.

This new species is named for its dense ribs, which distinguishes it from the most similar *S. albanicus*.

#### Distribution.

**Figure 7.**
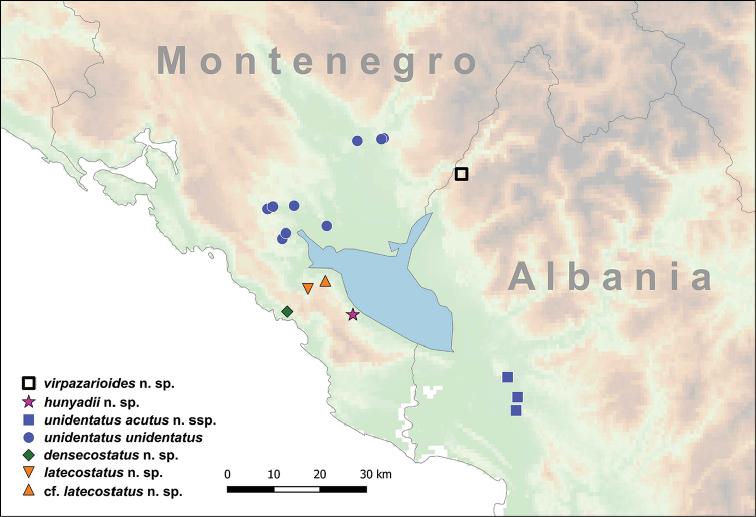
Distribution of *Spelaeodiscus* Brusina, 1886 species.

This species is known from the type locality only (Figure 7).

#### Conservation status.

The number of known locations of this species is less than five (i.e. known from a single site) and AOO is smaller than 20 km^2^, but there is no reason to suppose that AOO, EOO, number of locations, number of subpopulations or the number of mature individuals are declining or extremely fluctuating. Therefore, it should be assessed as Near Threatened (NT).

### Spelaeodiscus hauffeni

Taxon classificationAnimaliaStylommatophoraSpelaeodiscidae

(Schmidt, 1855)

Figure 8A–O

Helix Hauffeni Schmidt, 1855: 3–4.
Patula Hauffeni — Clessin 1887: 104.
Aspasita Hauffeni — Sturany and Wagner 1914: 67, plate 2, figs 11a–c.
Spelæodiscus hauffeni — Pilsbry 1926: 185, Plate 22, Figs 9–11.
Spelaeodiscus hauffeni — Bole 1965: 351–352, fig. 1b, 2a, Plate 76, fig. A.
Spelaeodiscus (Spelaeodiscus) hauffeni — Gittenberger 1969: 292–293, fig. 1.
Spelaeodiscus hauffeni — Welter-Schultes 2012: 213.

#### Type material.

**Figure 8.**
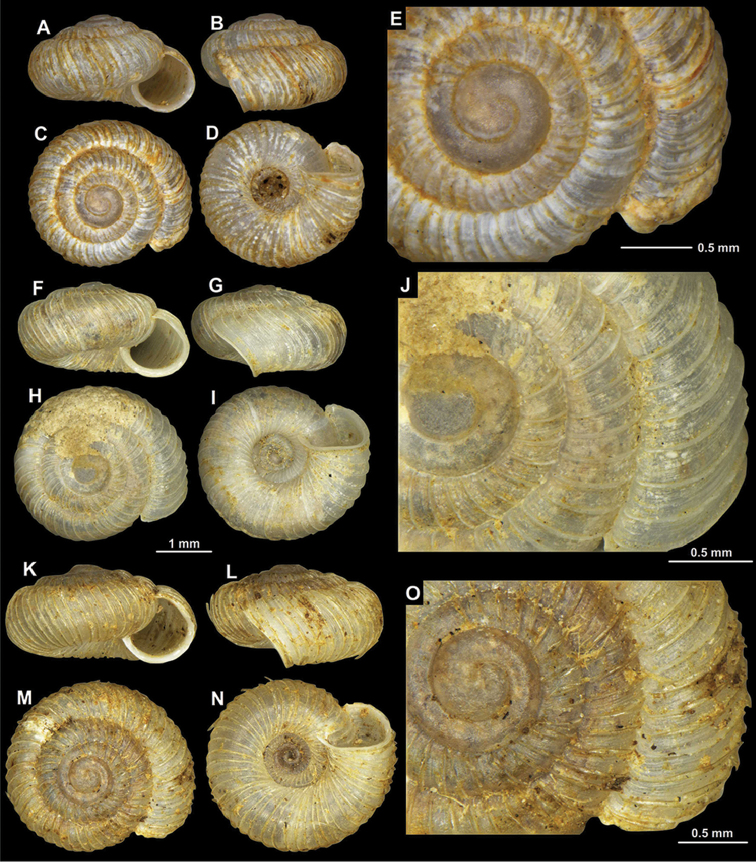
Shells of *Spelaeodiscus hauffeni* (Schmidt, 1855). **A–E** Syntype (NHMW 52776) **F–J** NHMW 71770/R/19 **K–O** Slovenia, Spodnja Kedenrica (NHMW 33672).

Krain: Krimberg-Grotte, ex coll. Schmidt, SMF 53902/3 syntypes; No locality, (“Orig. Ex.”), NHMW 52776/2 syntypes (Figure 8A–E).

#### Other material.

Velikajama, Soko Kod, Sela Dopilo (geographic position unknown), coll. Edlauer ex coll. Dabović, NHMW 48473/2 shells (1 adult + 1 juvenile) (in brackets: neben 49.999, probably mistyped 48.999, because this was a mixed lot of *S. hauffeni* and *S. dejongi*; this is obviously incorrect locality for this species); Jama nadjama pri Gnezdu (Izilovice) (= Jama pri Gnezdu at 45.939°N, 14.271°E), coll. Edlauer ex coll. Kuščer, NHMW 49137(?)/1 (incorrect locality); Krain, coll. Oberwimmer, NHMW 71640/O/00161/2; Carn., coll. Schmidt, NHMW 112346/2; Krain, coll. Kobelt ex coll. Ullepitsch, SMF 10663/2 adult+2 juvenile shells; Krain: Stubič, coll. C.R. Boettger, 1905, SMF 112878/1; Krainer Höhlen, coll. Kaltenbach ex coll. Müller, SMF 259470/1; Carniolia (= Krain), coll. Knobbe ex coll. Hauffen, SMF 53904/1; Höhle in Innerkrain, leg. Sever, coll. Ehrmann ex coll. Absolon, SMF 53905/1; Tekavičja jama (= Tkavčja jama, 45.824°N, 14.721°E), Dobrepolje, NHMW 112350/2 strongly corroded shells; Krain, Tekavcja jama, Debrepolje, coll. Edlauer ex coll. Kuščer, NHMW 48468/3; Tekarija jama b. Dobropolje, coll. S.H. Jaeckel ex coll. Kuščer, SMF 200961/3; Krain, Tekavčja jama, Dobrepolje, coll. Haas ex coll. Kuščer, SMF 53906/1; Krain, Berjakovo Brezno (= Malo Brezarjevo brezno, 46.080°N, 14.436°E), n. w. von Laibach, coll. Retaner (?), NHMW 12314/2; Berjakovo Brezno, nw v. Laibach, Krain, coll. Klemm ex coll. Edlauer, NHMW 79000/K/02938/2; Same data, coll. Schlickum, ex coll. Edlauer, SMF 275440/2; Berjakovo Brezno bei Dolnice n.w. von Ljubljana, coll. S.H. Jaeckel ex coll. Edlauer, SMF 200962/1; Radetina pećina, coll. Edlauer ex coll. Kuščer, NHMW 48751/6; Spodnja Skedevenica (at 45.854°N, 14.639°E), vel. Lašče, coll. Edlauer ex coll. Kuščer, NHMW 48883/15; Spodnja Skedevnica, coll. Edlauer ex coll. Kuščer, NHMW 48422/2; Spodnja Kedenrica, coll. W. Klemm ex coll. Kuščer, NHMW 33672/1 (figured shell, Fig. 8K–O); Sponja Skednevica bei Preserje, coll. Edlauer ex coll. Kuščer, NHMW 48124/2; Am Eingang der Doline Jama bei Kompole (=Kompoljska jama at 45.800°N, 14.731°E), S. Oest. von Dobrepolje, coll. Klemm, NHMW 112347/1; In der Raska Skedebnza bei Ponikve südl. von Dobrepolje (= Skedenc at 45.89687418°N, 14.50100919°E), coll. Klemm, NHMW 112348/3; Reoteiner Grotte bei Poduzik (?) bei Ljubljana (Kalkofen) (Podutiško brezno at 46.076°N, 14.445°E), coll. Edlauer ex coll. Kuščer, NHMW 49111/1; Brezarjeva brezno pri Boduzik (?), coll. Edlauer ex coll. Kuščer, NHMW 48390/2; Brezno v. Leskovi dolini (at 45.941°N, 14.256°E), coll. Edlauer ex coll. Kuščer, NHMW 49200/1; Mašun b Gratenbrunn (probably Jama na Mašunu at 45.630°N, 14.373°E), Krain, leg. Schollmayer, NHMW 112349/2 juvenile shells; Duplica (=Dupliška jama at 45.963°N, 14.684°E), coll. Edlauer ex coll. Kuščer, NHMW 48576/16; Jama v Jurčelovih(?) (Jama 1 v Jurcetovih Percah at 46.09984722°N, 14.42093707°E or Jama 2 na Jurcetovih Percah at 46.101°N 14.418°E, the first is indicated on the map), percah Tosko čelo, coll. Edlauer ex coll. Kuščer, NHMW 48688/16; Zakrita jama odd B (at 45.925°N, 14.293°E), coll. Edlauer ex coll. Kuščer, NHMW 48748/1 juvenile shell; Unterkrain: Grotte Thauzhia jama, coll. C. v. Heyden ex coll. N. Hoffmann, SMF 53903/1; Same data, coll. Gysser ex coll. Heynemann ex coll. C. v. Heyden, SMF 53901/2; Same data, coll. Heynemann ex coll. C. v. Heyden ex coll. N. Hoffmann, SMF 259469/3; Krain: Jeliarc-Grotte (maybe Jeliare or Jelice), coll. Th. Krüper, SMF 112877/1; Höhle Malo Bukuje bei Dobrova, Krain, coll. Dr. Leo Rušnov, ex coll. Dr. A. Oberwimmer, NHMW 71770/R/19 (1 photographed shell, Fig. 8F–J).

#### Diagnosis.

A medium sized species with very widely spaced, strong ribs, rounded, toothless aperture, and finely granular protoconch.

#### Description.

Spire somewhat elevated; protoconch consists of 1.5–1.75 whorls, very finely granulated, rather matte, not glossy; teleoconch with strong ribs that are supported by fine periostracal filaments in fresh shells; ribbing less regular than in other congeneric species; ribs widely spaced (41–52 ribs on body whorl); between main ribs some fine wrinkles discernible; entire shell with 3.75–4.25 whorls; aperture toothless, semilunar/rounded; peristome slightly thickened, slightly reflected in direction of umbilicus; umbilicus regular funnel-shaped, relatively narrow.

**Measurements.** SW: 2.8–3.5 mm (median = 3.0 mm), SH: 1.7–1.9 mm (median = 1.8 mm), AW: 1.1–1.4 mm (median = 1.2 mm), AH: 1.0–1.3 mm (median = 1.1 mm), (n = 6; largest and smallest specimens of multiple populations measured).

#### Differential diagnosis.

The most similar taxon to *Spelaeodiscus hauffeni* is *S. albanicus edentatus* ssp. n. in terms of shell size, shape, and rib density. However, the latter one is usually larger, has a glossier protoconch, a less rounded aperture caused by the straighter basal part, and the peristome edge on the palatal side is more strongly expanded (rather thickened only in *S. hauffeni*).

#### Variation among specimens.

*Spelaeodiscus hauffeni* shows some variability in terms of shell size and spire height, but the rib density and the formation of the aperture are stable characters.

#### Distribution.

**Figure 9.**
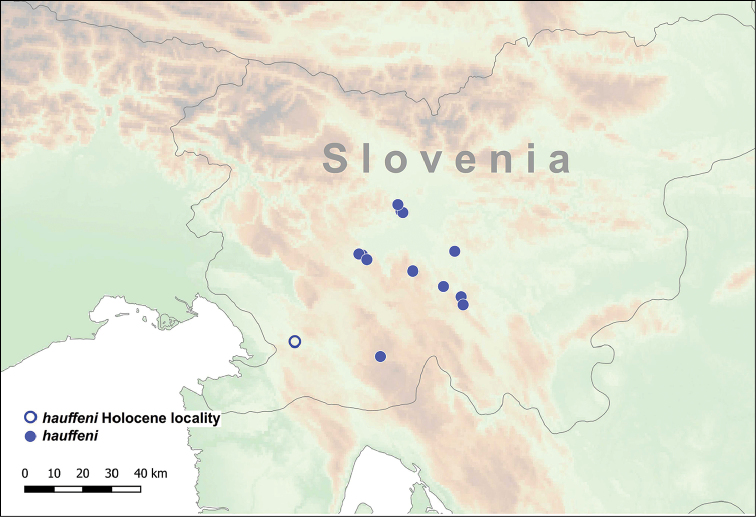
Distribution of *Spelaeodiscus hauffeni* (Schmidt, 1855).

This species is distributed in the southeastern Alps (Central Slovenia) relatively far from the ranges of its congeneric taxa. Slapnik (2005) found shells in the Škocjan Caves Regional Park, which are probably of Holocene age (Figure 9).

#### Conservation status.

As there are several known locations and no reason to suppose that the habitat quality, habitat extent or population are deteriorating or extremely fluctuating, it was assessed as Least Concern (LC) by Reischütz (2017b).

### Spelaeodiscus hunyadii

Taxon classificationAnimaliaStylommatophoraSpelaeodiscidae

Páll-Gergely & Deli sp. n.

http://zoobank.org/5DB55567-0640-4C82-8BF3-B2A281366C7A

Figure 10A–E

#### Type material.

**Figure 10.**
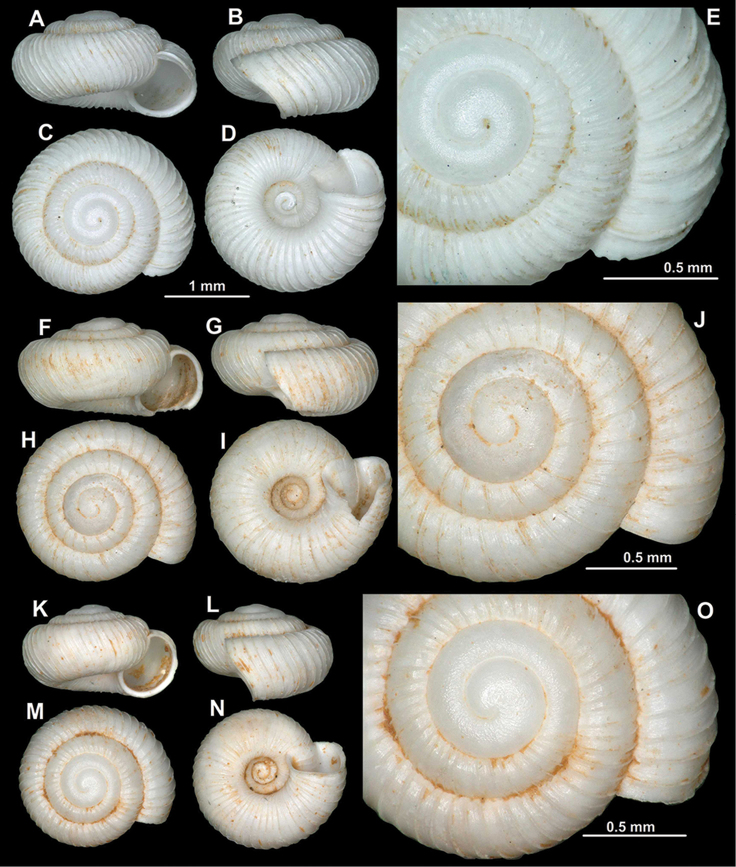
Shells of *Spelaeodiscus* Brusina, 1886 species. **A–E** *Spelaeodiscus hunyadii* sp. n., holotype (HNHM 103210) **F–J** *Spelaeodiscus latecostatus* sp. n., holotype (HNHM 103214) **K–O** Spelaeodiscus cf. latecostatus sp. n., Montenegro, Seoća S 1 km (HNHM 103213).

Montenegro, SE of Virpazar, 4.3 km (in a straight line) SSE of Ðuravci, near Besa/Bes near Krone i Besit, 330 m a.s.l., 42°08.548'N, 19°13.165'E (site code: 20171019G), leg. T. Deli, Z.P. Erőss, A. Hunyadi & B. Páll-Gergely, 19.10.2017, HNHM 103210 (holotype, SW: 2.1 mm, SH: 1.2 mm, Fig. 10A–E), DT/40 paratypes, EZP/40 paratypes, HA/40 paratypes, HNHM 103211/3 paratypes, PGB/40 paratypes, NMBE 554178/3 paratypes, NHMW 112367/4 paratypes; Montenegro, Donji Murići junction S 2 km along the Virpazar-Ostros road, 320 m a.s.l., 42°8.544'N, 19°13.182'E (site code: 2017/009), leg. Z.P. Erőss & Z. Fehér, 16.07.2017, HNHM 103212/3 paratypes, EZP/3 paratypes.

#### Type locality.

Montenegro, SE of Virpazar, 4.3 km (in a straight line) SSE of Ðuravci, near Besa/Bes near Krone i Besit, 330 m a.s.l., 42°08.548'N, 19°13.165'E.

#### Diagnosis.

A small, nearly flat species with strong, widely spaced ribs, glossy protoconch and strongly oblique, toothless aperture.

#### Description.

Spire somewhat elevated; protoconch consists of 1.25–1.5 whorls, smooth, glossy; teleoconch with very strong (thick), equidistant, widely spaced ribs; rib density: 42–48 ribs on body whorl; between main ribs some fine wrinkles discernible; entire shell with 3.5–3.75 whorls; aperture semilunar, toothless, strongly oblique to shell axis; peristome slightly thickened and expanded in direction of umbilicus; umbilicus funnel-shaped, relatively narrow.

**Measurements.** SW: 2.1–2.2 mm (median = 2.2 mm), SH: 1.2–1.3 mm (median = 1.2 mm), AW: 0.8–0.9 mm (median = 0.9 mm), AH: 0.6–0.7 mm (median = 0.7 mm), AA = 47–52°(n = 4; largest and smallest specimens measured).

#### Differential diagnosis.

*Spelaeodiscus obodensis* has a more conical shell and roughly sculptured protoconch. The most similar species to *Spelaeodiscus hunyadii* sp. n. is *S. dejongi*, which lives sympatrically with the new species. It differs from *Spelaeodiscus hunyadii* sp. n. in the less oblique aperture and the denser ribs. Some other populations of *S. dejongi* also possess widely spaced ribs, but their aperture is less oblique, thus, they can be distinguished from this new species. See also under *S. latecostatus* sp. n.

#### Variation among specimens.

Specimens of the type sample show no notable conchological variability.

#### Etymology.

This new species is named after our colleague and friend, András Hunyadi, who is one of those who first collected this species.

#### Distribution.

This species is known from the type locality only (Figure 7).

#### Conservation status.

To our present knowledge this species is very rare (currently known from a single location) and thus AOO is smaller than 20 km^2^. However, there is no reason to suppose that AOO, EOO, number of locations, number of subpopulations or the number of mature individuals are declining or extremely fluctuating. Therefore, it might be assessed as Near Threatened (NT).

### Spelaeodiscus latecostatus

Taxon classificationAnimaliaStylommatophoraSpelaeodiscidae

Páll-Gergely & Erőss sp. n.

http://zoobank.org/AEFE6B2C-573F-4C04-8353-4A413B536F24

Figure 10F–O

#### Type material.

Montenegro, S of Virpazar, 0.8 km (in a straight line) E of Limljani, above the village, 400 m a.s.l., 42°11.698'N, 19°06.217'E (site code: 20171021B), leg. T. Deli, Z.P. Erőss, A. Hunyadi & B. Páll-Gergely, 21.10.2017, HNHM 103214 (holotype, Fig. 10A–E).

#### Other material.

Montenegro, Seoća S 1 km, along the Virpazar-Ostros road, 280 m a.s.l., 42°12.618'N, 19°9.000'E (site code: 2017/005), leg. Z.P. Erőss & Z. Fehér, 16.07.2017, HNHM 103213/3 shells, not paratypes (Fig. 10K–O).

#### Type locality.

Montenegro, S of Virpazar, 0.8 km (in a straight line) E of Limljani, above the village, 400 m a.s.l., 42°11.698'N, 19°06.217'E (site code: 20171021B).

#### Diagnosis.

A small, nearly flat species with strong, very widely spaced ribs, glossy protoconch and a toothless aperture.

#### Description of the holotype.

Spire somewhat elevated; protoconch consists of ca 1.25–1.5 whorls (the holotype is corroded at the protoconch-teleoconch junction), rather smooth, moderately glossy; teleoconch with very strong, equidistant, widely spaced ribs (42 on the body whorl); between main ribs some fine wrinkles discernible; entire shell with 3.75 whorls; aperture semilunar, toothless; peristome slightly thickened and expanded; umbilicus funnel-shaped, relatively narrow.

**Measurements.** SW: 2.2 mm, SH: 1.2 mm, AW: 0.8 mm, AH: ca. 0.8 mm, AA = 63°(holotype).

#### Differential diagnosis.

The widely spaced ribs are similar to *S. hunyadii* sp. n., but the less oblique aperture distinguishes *S. latecostatus* sp. n. from the other new species. *Spelaeodiscus dejongi*, which lives sympatrically with *S. latecostatus* sp. n., is similar in shell shape and size and the formation of the aperture, but has much denser ribs.

#### Variation among specimens.

See remarks.

#### Etymology.

This new species is named after its remarkably widely spaced ribs.

#### Distribution.

See under Remarks and Figure 7.

#### Remarks.

The holotype of this species was found in a large sample of *S. dejongi*. Therefore, even if the shell shape does not differ from that species, the widely spaced ribs indicate that *S. latecostatus* sp. n. differs from *S. dejongi* on species level. Three shells from 1 km S of Seoća possess denser ribs than other *S. dejongi* populations (47–54 ribs on the body whorl), but obviously the rib density is lower than that of the holotype of *S. latecostatus* sp. n. Since the rib density of that population is intermediate between *S. dejongi* and *S. latecostatus* sp. n., it is not possible to decide which species it belongs to. More populations around the sample from 1 km S of Seoća site are necessary in order to provide a reliable identification. Here we provisionally identify those shells as Spelaeodiscus cf. latecostatus sp. n.

#### Conservation status.

To our present knowledge this species is very rare (currently known from two locations) and thus AOO is smaller than 20 km^2^. However, there is no reason to suppose that AOO, EOO, number of locations, number of subpopulations or the number of mature individuals are declining or extremely fluctuating. Therefore, it might be assessed as Near Threatened (NT).

### Spelaeodiscus obodensis

Taxon classificationAnimaliaStylommatophoraSpelaeodiscidae

Bole, 1965

Figures 11 , 12

Spelaeodiscus obodensis Bole, 1965: 350, plate 76, fig. D.
Spelaeodiscus (Spelaeodiscus) obodensis — Gittenberger 1969: 296–297, fig. 1.
Spelaeodiscus obodensis — Welter-Schultes 2012: 213. (partim: the photos show S. unidentatus specimens)

#### Type material.

Obodska pećina, Rij. Crnojevića, Mtg., September 1956, MZBI 1018 (2 adult and 3 juvenile/broken syntypes, photos were examined, Figure 12A).

#### Material examined.

**Figure 11.**
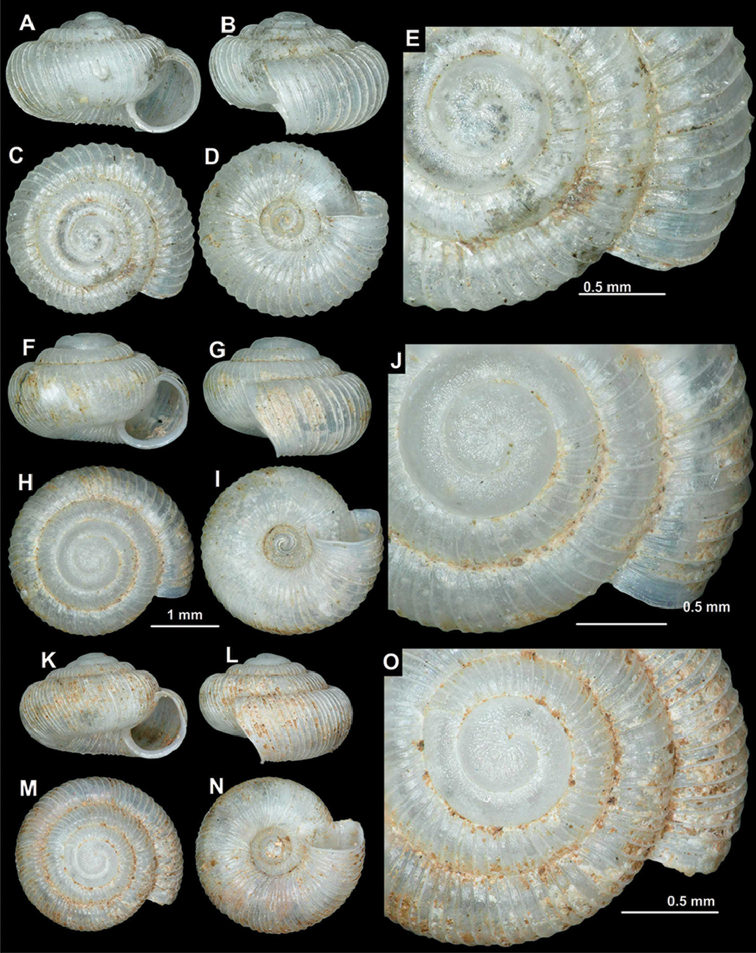
*Spelaeodiscus obodensis* Bole, 1965. **A–E** Pecina u Peckom Brdu cave, above Zacir (HNHM 18062) **F–J** Montenegro, Ostrog, entrance of the penultimate parking lot (NHMW 112365) **K–O** Montenegro, 500 m south of the bridge over the Sitnica river (NHMW 112366).

Vodna jama (Lovćen), coll. Edlauer ex coll. Kuščer, NHMW 48543/2 (det. Gittenberger, 1973 Sep.); Same data, NHMW 49765/2; Montenegro, Ostrog, rocks below the upper parking lot, 820 m a.s.l., 42°40.534'N, 19°1.744'E, leg. A. Reischütz, P. L. Reischütz & N. Steiner-Reischütz, May 2017, REI/1; Montenegro, Ostrog, entrance of the penultimate parking lot, 770 m a.s.l., 42°40.510'N, 19°1.663'E, leg. A. Reischütz, P. L. Reischütz & N. Steiner-Reischütz, May 2017, NHMW 112365 (photographed shell, Fig. 11F–J), REI/1; Montenegro, Ostrog, 1 km north from Hotel Sokoline on the new street to Podgorica, 630 m a.s.l., 42°39.498'N, 19°2.188'E, leg. A. Reischütz, P. L. Reischütz & N. Steiner-Reischütz, May 2017, REI/2; Montenegro, 500 m south of the bridge over the Sitnica river, north of Podgorica, 45 m a.s.l., 42°27.473'N, 19°10.825'E, leg. A. Reischütz, N. Reischütz & P. L. Reischütz, Apr. 2012, NHMW 112366/1 (photographed shell, Fig. 11K–O), REI/2; Yugoslavia: Pečina u Peckom Brdu bg., above Začir, leg. L. Pintér & P. Subai, 20 Jul. 1972, HNHM 41127/2; Same data, HNHM 18062/3 (one of them is photographed, Fig. 11A–E); Montenegro, Pečina u peckom Brdu bei Začir (Brdu-cave at Začir), leg. H. Schütt, 06.06.1978, HNHM 42402/3.

#### Diagnosis.

A medium sized species with elevated spire, roughly sculptured protoconch, strong ribs on the teleoconch, and toothless aperture.

#### Description.

Spire elevated, shell low conical; protoconch consists of 1.25–1.5 whorls, roughly granulated/”hammered”, matte, not glossy; teleoconch with strong, equidistant ribs that are supported by fine periostracal filaments in fresh shells; rib density variable (43–76 ribs on body whorl), usually widely spaced; between main ribs some fine wrinkles discernible; entire shell with 3.75–4 whorls; aperture semilunar, toothless; peristome slightly thickened and expanded, especially in direction of the umbilicus; umbilicus funnel-shaped, relatively narrow.

**Measurements.** SW: 2.6–3.0 mm (median = 2.8 mm), SH: 1.6–1.9 mm (median = 1.7 mm), AW: 0.9–1.2 mm (median = 1.1 mm), AH: 1.0–1.2 mm (median = 1.1 mm), AA = 64–70°(n = 10; largest and smallest specimens of multiple populations measured).

#### Differential diagnosis.

The most similar species is *S. dejongi*, which usually has a lower spire, weaker ribs, and a glossy protoconch.

#### Variation among specimens.

This species is the most variable in terms of shell size and rib density.

#### Distribution.

This species is found northwest of the Shkodër Lake Basin, as well as in the Zeta River Valley between Podgorica and the Ostrog Monastery (Figure 4). The Albanian record given by Reischütz et al. (2013) and referred by Reischütz (2017c) is actually *S. albanicus edentatus* ssp. n. So far, no Albanian occurrence is known.

#### Conservation status.

As there are at least seven known locations and no reason to suppose that the habitat quality, habitat extent or population are deteriorating or extremely fluctuating, it was assessed as Least Concern (LC) by Reischütz (2017c).

**Figure 12.**
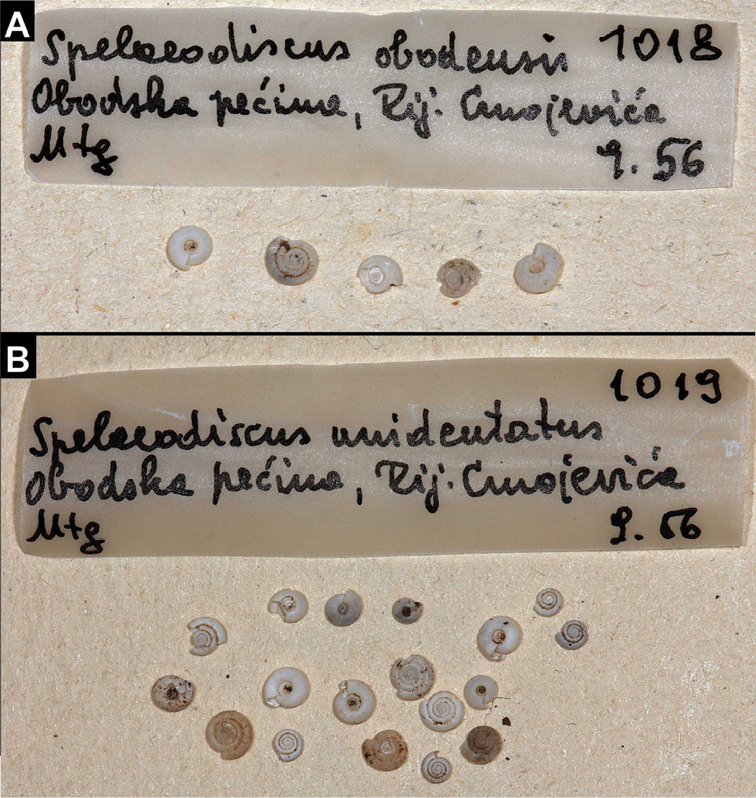
Type sample of *Spelaeodiscus obodensis* Bole, 1965 (**A**) and *Spelaeodiscus unidentatus unidentatus* Bole, 1961 (**B**).

### Spelaeodiscus unidentatus

Taxon classificationAnimaliaStylommatophoraSpelaeodiscidae

Bole, 1961

#### Diagnosis.

A small to medium sized species with dense riblets, and strong parietal and basal teeth/thickenings.

#### Differential diagnosis.

This species differs from most other *Spelaeodiscus* species by the presence of two well-developed apertural teeth (a palatal and a basal). See also under *Spelaeodiscus albanicus*.

#### Conservation status.

As there are more than five known locations and no reason to suppose that the habitat quality, habitat extent or population are deteriorating or extremely fluctuating, it was assessed as Least Concern (LC) by Reischütz (2017d).

### Spelaeodiscus unidentatus unidentatus

Taxon classificationAnimaliaStylommatophoraSpelaeodiscidae

Bole, 1961

Figure 13

Spelaeodiscus unidentatus Bole, 1961: 205 fig. 1a–d.
Spelaeodiscus unidentatus — Bole 1965: 350–351, fig. 1a, 2b, plate 76, fig. C.
Spelaeodiscus (Spelaeodiscus) unidentatus — Gittenberger 1969: 297, fig. 1.
Spelaeodiscus unidentatus — Welter-Schultes 2012: 214.

#### Type material.

Obodska pećina, Rij. Crnojevića, Mtg., 1956 September, MZBI 1019/6 adult syntypes and some juvenile syntypes (photos of the sample where examined, Figure 12B). See also Remarks.

#### Other material.

**Figure 13.**
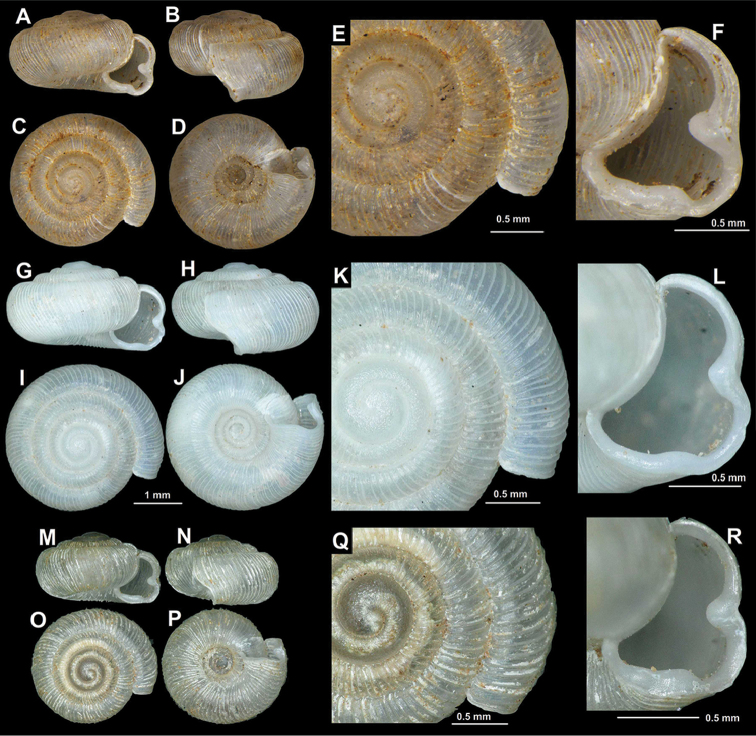
*Spelaeodiscus unidentatus unidentatus* Bole, 1961. **A–F** Pecina u Peckom Brdu cave, above Zacir (HNHM 65187, topotypic specimen) **G–L** Montenegro, Morača valley, after the exit to the “China Road and Bridge” camp N of Bioće (NHMW 112368) **M–R** Montenegro, spring 1 km south of the junction towards Njive (NHMW 112369).

Bioče, N Titograd, Mtg. 23.09.1978, MZBI 15443 (photos of the sample where examined); Megara, Toluši, Titograd, Mtg. MZBI 2335 (photos of the sample where examined); Pećina od Zavora, Peuta, Titograd, Mtg. 01.11.1963, MZBI 2338 (photos of the sample where examined); Höhle bei Virpazar, leg. Dabović, coll. Edlauer, NHMW 48881/1; Brošine cave (33), leg. Kuscer 10266/67, coll. Edlauer, NHMW 38874/a (20 juvenile shells, “schlechte Expl”); Same data, NHMW 58874/4 (“bessere Expl”); Virpazar, mit “oculus mundi”, leg. Dabović, coll. Edlauer, NHMW 78988/3 juvenile shells (det. Gittenberger, but identification not certain); Smarjetna gora + Grabočica cave (drawing of a cave on the label), (Trnovo), NHMW 78989/7; Same data, NHMW 48086/5; Vodna jama (Lovćen), coll. Edlauer ex coll. Kuščer, NHMW 48544/1; Same locality, coll. Edlauer, NHMW 49764/1 (labelled as “*Spelaeodiscus albanicus hadzii*”); Montenegro, NNW of Virpazar, road between Rijeka Crnojevića and Virpazar, 0.4 km (in a straight line) W of Poseljani, 130 m a.s.l., 42°18.361'N, 19°02.898'E (site code: 20171020F), leg. T. Deli, Z.P. Erőss, A. Hunyadi & B. Páll-Gergely, 20.10.2017, DT/ca. 15, EZP/ca. 15, HA/ca. 15, HNHM 103248/5, PGB/ca. 15; Montenegro, W of Rijeka Crnojevića, 1 km (in a straight line) NNE of Zaćir, above Obodska pećina, near the road, 320 m a.s.l., 42°21.168'N, 19°00.125'E (site code: 2017.10.20J), leg. T. Deli, Z.P. Erőss, A. Hunyadi & B. Páll-Gergely, 20.10.2017, DT/ca. 18, EZP/ca. 18, HA/ca. 18, HNHM 103249/5, PGB/ca. 18; Montenegro, 2 km north of Velje Brdo in the Zeta valley, north of Podgorica, 70 m a.s.l., 42°28.932'N, 19°14.454'E, leg. A. Reischütz, P. L. Reischütz & N. Steiner-Reischütz, May 2016, REI/5; Montenegro, Morača valley, after the exit to the “China Road and Bridge” camp N of Bioće, 70 m a.s.l., 42°29.167'N, 19°18.646'E, leg. A. Reischütz, P. L. Reischütz & N. Steiner-Reischütz, May 2016, NHMW 112368/1 (photographed shell, Fig. 13G–L), REI/10; Montenegro, road between Rijeka Crnojevića and Hrvasi, 180 m a.s.l., 42°21.491'N, 19°4.281'E, leg. A. Reischütz, N. Reischütz & P. L. Reischütz, Apr. 2012, REI/11; Montenegro, scree slope at the BMS-petrol station, south of the Morača gorge, 5 km south of Bioće, 90 m a.s.l., 42°29.064'N, 19°18.279'E, leg. A. Reischütz, N. Reischütz & P. L. Reischütz, Jul. 2008, REI/1; Montenegro, spring 1 km south of the junction towards Njive, south of Rijeka Crnojevića, 150 m a.s.l., 42°18.266'N, 19°2.900'E, leg. A. Reischütz, N. Reischütz & P. L. Reischütz, Mar. 2011, NHMW 112369/1 (photographed shell, Fig. 13M–R); Montenegro, Žabljak Crnojevića, fortress, 50 m a.s.l., 42°19.030'N, 19°9.401'E (site code: 2015/007), leg. T. Deli, Z.P. Erőss & Z. Fehér, 25.05.2015, HNHM 103250/1 adult and several juvenile shells; Montenegro, Začir, “Pečina u pecko brdo” (cave), 450 m a.s.l., leg. L. Pintér, P. Subai, A. Szigeti, 20 Jul. 1972, NMBE 542072/76; Montenegro, Pečina u peckom, Brdu-bg Začirnál (Brdu-cave at Začir), leg. L. Pintér & P. Subai, 20 Jul. 1972, HNHM 65187/7 (one of them is photographed: Figs 13A–F); Montenegro, Pečina u peckom Brdu bei Začir (Brdu-cave at Začir), leg. H. Schütt, 06.06.1978, coll. Gy. Kovács, HNHM 65188/1; Same data, HNHM 42401/2; Montenegro, Rijeka Crnojevića, Obodska cave, 110 m a.s.l., 42°21.120'N, 19°0.318'E, leg. P. Subai & M. Szekeres, 23 Sep. 2006; NMBE 542071/1; Montenegro, Poseljani, on the Virpazar–Rijeka Crnojevića road, 150 m a.s.l., 42°18.347'N, 19°2.884'E (site code: 2015/016), leg. T. Deli, Z.P. Erőss & Z. Fehér, 25.05.2015, HNHM 103251/8; NHMW 112370/8, DT/8.

#### Diagnosis.

Basal tooth/thickening low, not pointed; palatal part of peristome with strong incision at the position of the palatal tooth, palatal region strongly “pushed” from outside.

#### Description.

Spire elevated; protoconch consists of 1.5 whorls, roughly granulated/”hammered”, matte, not glossy; teleoconch with strong but dense, equidistant ribs that are supported by fine periostracal filaments in fresh shells; rib density variable (74–118 ribs on body whorl); between main ribs some fine wrinkles discernible; entire shell with 3.75–4.5 whorls; aperture semilunar or triangular due to straight basal part; peristome thickened, slightly expanded and slightly reflected on the palatal, basal and umbilical areas; palatal tooth strong, pointed; palatal region of peristome with strong outer incision (i.e. the position of the palatal tooth is indicated with a groove on the outer side); basal portion of peristome straight, thickened; occasionally two small denticles visible, sometimes only the one situated closer to the palatal side is developed as a low denticle; umbilicus funnel-shaped, wide to relatively narrow (width depends on spire height).

**Measurements.** SW: 2.4–3.2 mm (median = 2.7 mm), SH: 1.3–1.8 mm (median = 1.5 mm), AW: 0.9–1.1 mm (median = 1.0 mm), AH: 1.0–1.4 mm (median = 1.1 mm), (n = 11; largest and smallest specimens of multiple populations measured).

#### Differential diagnosis.

See under *S. unidentatus acutus* ssp. n.

#### Variation among specimens.

This subspecies is particularly variable in terms of shell size. The shape of the thickening of the basal part of the peristome is also slightly variable, although it never develops to a pointed tooth.

#### Distribution.

This taxon is found north and northwest of the Shkodër Lake Basin in Montenegro (Figure 7). Albanian records given by Reischütz et al. (2013) actually refer to *S. unidentatus acutus* ssp. n.

#### Remarks.

The only sample in the MZBI, which was collected before the original description is from Obodska pećina represent the type sample of this species (MZBI 1019). Bole collected both *S. obodensis* and *S. unidentatus* at Obodska pećina in September, 1956, but described only one (*S. unidentatus*) in 1961, and the other (*S. obodensis*) in 1965.

Formerly, Peuta Cave population was incorrectly referred to as *S. albanicus* and was taken into consideration in that species’ Red List assessment (Reischütz and Fehér 2017).

### Spelaeodiscus unidentatus acutus

Taxon classificationAnimaliaStylommatophoraSpelaeodiscidae

Páll-Gergely & Fehér ssp. n.

http://zoobank.org/90A2874A-9BE4-474F-A572-8A1FC36B6172

Figure 14A–L

Spelaeodiscus unidentatus — Reischütz et al. 2013: 62, fig. 3.

#### Type material.

**Figure 14.**
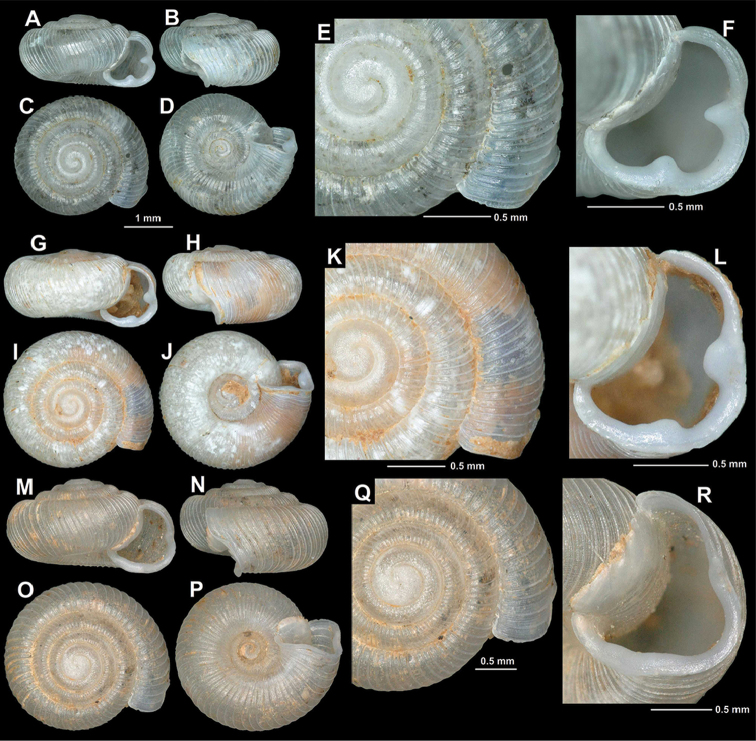
Shells of *Spelaeodiscus*. **A–F** *Spelaeodiscus unidentatus acutus* ssp. n., holotype (HNHM 103252) **G–L** *Spelaeodiscus unidentatus acutus* ssp. n., Albania, rocks north of Hajmel at the bridge (NHMW 112371) **M–R** *Spelaeodiscus virpazarioides* sp. n., holotype (HNHM 103417).

Albania, rocks north of Hajmel at the bridge, north of Lezhe, 40 m a.s.l., 41°58.426'N, 19°38.683'E, leg. A. Reischütz, N. Reischütz & P. L. Reischütz, Apr. 2012, NHMW 112371/1 (photographed paratype, Fig. 14G–L), REI/6 paratypes; Albania, rocks 1 km south of Hajmel, north of Lezhe, 50 m a.s.l., 41°56.848'N, 19°38.379'E, leg. A. Reischütz, N. Reischütz & P. L. Reischütz, Apr. 2012, REI/5 paratypes; Albania, Shkodër District, Vau i Dejës, Mjedë, near the dam, 45 m a.s.l., limestone rocks, 42°0.804'N, 19°37.188'E (site code: 2015/99), leg. Z.P. Erőss, Z. Fehér & J. Grego, 04 Jul. 2015, HNHM 103252 (holotype, SW: 2.9 mm, SH: 1.5 mm, Figs 14A–F), NHMW 112372/1 paratype, JG/1 paratype.

#### Type locality.

Albania, Shkodër District, Vau i Dejës, Mjedë, near the dam, 45 m a.s.l., limestone rocks, 42°0.804'N, 19°37.188'E.

#### Diagnosis.

Basal tooth pointed; palatal part of peristome with slight incision at the position of the palatal tooth, palatal region not or slightly “pushed” from outside.

**Measurements.** SW: 2.9–3.5 mm (median = 3.0 mm), SH: 1.4–1.8 mm (median = 1.5 mm), AW: 1.1–1.3 mm (1.2 mm), AH: 1.1–1.4 mm (median = 1.2 mm), (n = 9; largest and smallest specimens of multiple populations measured).

#### Differential diagnosis.

*Spelaeodiscus unidentatus acutus* ssp. n. differs from the nominotypical subspecies by the strong, pointed basal tooth, which is blunt, or represented as a thickening of the basal peristome in the nominotypical subspecies. Moreover, the palatal region is not or only slightly “pushed” from the outside in the position of the palatal tooth. Rib density: 64–91 ribs on the body whorl.

#### Variation among specimens.

This subspecies is variable in terms of shell size and spire height, i.e. one population has a nearly flat shell.

#### Etymology.

This new subspecies is named after its pointed (Latin: *acutus*) basal tooth, which distinguishes it from the nominotypical subspecies.

#### Distribution.

This subspecies is known from the southeastern side of the Shkodër Lake Basin in Albania (Figure 7).

### Spelaeodiscus virpazarioides

Taxon classificationAnimaliaStylommatophoraSpelaeodiscidae

Páll-Gergely & Fehér sp. n.

http://zoobank.org/6454FD89-D6C1-4607-9992-7F1CC7DBD617

Figure 14M–R

#### Type material.

Albania, Malësia district, a mountain pass 2 km N of Rraps-Starjë, 700 m a.s.l., 42°24.888'N, 19°30.240'E, leg. Erőss, Fehér, Szekeres, Grego, 27.06.2016, HNHM 103417 (holotype, SW = 3.45 mm, SH = 2.0 mm), HNHM 102765/4+1subadult+3fr (paratypes), NHMW 111672/3+2subadult+2fr; JG/3+2subadult+3fr (paratypes); same locality, leg. A. Reischütz, N. Reischütz & P. Reischütz, Apr. 2006, REI/1 juvenile paratype.

#### Type locality.

Albania, Malësia district, a mountain pass 2 km N of Rraps-Starjë, 700 m a.s.l., 42°24.888'N, 19°30.240'E.

#### Diagnosis.

A medium sized to large species with elevated spire, strong, very widely spaced ribs, fine spiral lines consisting of series of minute tubercles, hammered protoconch, weak basal thickening, weak parietal tooth, and a thickened parietal callus.

#### Description.

Spire elevated; protoconch consists of ca. 1.25–1.75 whorls, roughly sculptured, “hammered”; teleoconch with very strong, equidistant, widely spaced ribs (40–70 on the body whorl; addition to the radial ribs very fine, dense spiral striation is visible on the entire teleoconch, consisting of minute tubercles; entire shell with 4–4.75 whorls; aperture subcircular/triangular; basal part with two low swellings (similar to those of *S. albanicus albanicus*), parietal wall with a weak tooth; parietal callus thickened, blunt; umbilicus funnel-shaped, relatively narrow.

**Measurements.** SW: 3.3–3.6 mm (median = 3.5 mm), SH: 1.95–2.25 mm (median = 2.1 mm), AW: 1.3–1.6 mm (median = 1.4 mm), SW: 1.4–1.5 mm (median = 1.4 mm), (n = 11).

#### Differential diagnosis.

This new species can be distinguished from all congeners (especially the most similar *S. albanicus albanicus*) by the clearly visible spiral striation, roughly sculptured protoconch and thickened parietal callus. All *Virpazaria* species possess an elevated parietal callus, but it is sharp in most (all?) species, and the aperture of those species are more slender, crescent-shaped.

#### Variation among specimens.

The degree of the thickness of the parietal callus shows some recognisable variability within the single known population, but this trait might be due to the age (degree of development) of the examined shells.

#### Etymology.

This new species is named after its resemblance to *Virpazaria* species based on the thickened parietal callus.

#### Distribution.

*Spelaeodiscus virpazarioides* sp. n. is known from the type locality only (see also Figure 7).

#### Remarks.

Initially we considered placing this species to the genus *Virpazaria* due to the thickened parietal callus, which was mentioned in the original description of the genus *Virpazaria*. However, the subcircular/triangular aperture indicates that it is better to be placed in *Spelaeodiscus*.

Formerly, this population was incorrectly referred to as *S. albanicus* and was taken into consideration in that species’ Red List assessment (Reischütz and Fehér 2017)

#### Conservation status.

To our present knowledge this species is very rare (currently known from a single location) and thus AOO is smaller than 20 km^2^. However, there is no reason to suppose that AOO, EOO, number of locations, number of subpopulations or the number of mature individuals are declining or extremely fluctuating. Therefore, it might be assessed as Near Threatened (NT).

## Discussion

### Species recognition and biogeography

We examined all available *Spelaeodiscus* samples from the Western Balkan area (Slovenia, Montenegro, Albania). Our main aim was to delimit (sub)species based on conchological characters. We found that shell size, spire height and rib density is particularly variable within and between populations, and can be used only with caution for species recognition. Morphology of ribs, however (elevated or low, thin, lamella-like or strong, calcareous), the fine sculpture of the protoconch (glossy or granulate), and the obliqueness of the aperture to the shell axis are useful character in several cases. The two subspecies described here (*S. albanicus edentatus* ssp. n. and *S. unidentatus acutus* ssp. n.) are primarily recognized based on differences of the apertural teeth. The most important traits distinguishing the species delimited here are hardly or not quantifiable. Thus, no statistical tests are applied to verify our taxonomic decisions. We are aware of the limitations of the exclusively conchological approach, especially in case of species known from single shells or single populations. However, given the commonness of rarity, omitting the description of singletons would prevent the description of a very significant proportion of the species-level diversity (Lim et al. 2012).

*Spelaeodiscus dejongi* has the largest known area and is known from most numerous populations. In face of the large variability between populations we found no qualitative traits that would distinguish populations on species or subspecies level, therefore we treat it as a single, variable species. However, future studies should focus on the degree of differences on molecular level.

Although the majority of species is known from multiple populations of relatively large areas, three single-site endemic species (*S. densecostatus* sp. n., *S. hunyadii* sp. n., *S. latecostatus* sp. n.) are also described. The latter two new species were found in sympatry with the widely distributed *S. dejongi*, which is an evidence that none of them are conspecific with *S. dejongi* (see Table 2). More precise field collections should focus on the area they inhabit in order to examine the true extent of their range.

**Table 2.** Co-occurrences of *Spelaeodiscus* species.

Locality	co-occurring species
Montenegro, SE of Virpazar, 4.3 km (in a straight line) SSE of Ðuravci, near Besa/Bes near Krone i Besit, 330 m a.s.l.	*S. hunyadii* sp. n. & *S. dejongi*
Yugoslavia: Pečina u Peckom Brdu cave above Začir	*S. unidentatus unidentatus* & *S. obodensis*
Obodska pećina, Rij. Crnojevića	*S. unidentatus unidentatus* & *S. obodensis*
Montenegro, S of Virpazar, 0.8 km (in a straight line) E of Limljani, above the village, 400 m a.s.l.	*S. latecostatus* sp. n. & *S. dejongi*

### Habitat

*Spelaeodiscus* (in contrast to *Aspasita*) is obviously a subterranean genus. Up to the recent past, only a few populations were known and it was believed to be troglobiont, because – if not in fluvial flotsam – all findings were from or near caves. In the past decade, some other gastropod taxa, previously believed to be troglobiont, turned up in non-carbonate areas, or in limestone but assuredly outside of caves (Deli and Subai 2011, Subai 2011, Dedov 2015; Reischütz et al. 2016). These findings already demonstrated that *Agardhiella* Hesse, 1923, *Gyralina* Andreae, 1902, *Sciocochlea* C.R. Boettger, 1935 and *Tsoukatosia* Gittenberger, 2000 have affinity to the superficial underground compartments (also known as “Mesovoid Shallow Substratum” or “Milieu Souterrain Superficiel”, widely abbreviated as MSS, see e.g. Juberthie et al. 1980, 1981; Camacho 1992; Culver and Pipan 2009, Mammola et al. 2016).

We have collected all *Spelaeodiscus* samples in fissures or small cavities of bare limestone cliffs, from which fine granulated material could be yielded. Although we have found only empty shells, and therefore it is still not completely clear where they actually live, the abundance of *Spelaeodiscus* in these superficial fissures seems to be larger than in cave deposits. This prompted us to question whether *Spelaeodiscus* is truly troglobiont.

Literature data are very scarce about MSS dwelling gastropods, and still, that little is dealing mostly with scree slopes, habitats where fragments of rocks are accumulating at the bottom of rocky walls, and are covered over time by an evolving soil (Arndt and Subai 2013, Rendoš et al. 2014). Scree slopes were reported to harbour exclusively edaphic species, but Arndt and Subai (2013) presume that this was due to the applied passive sampling method (buried pitfall traps), which is ineffective for catching small sized and immobile snails.

**Figure 15.**
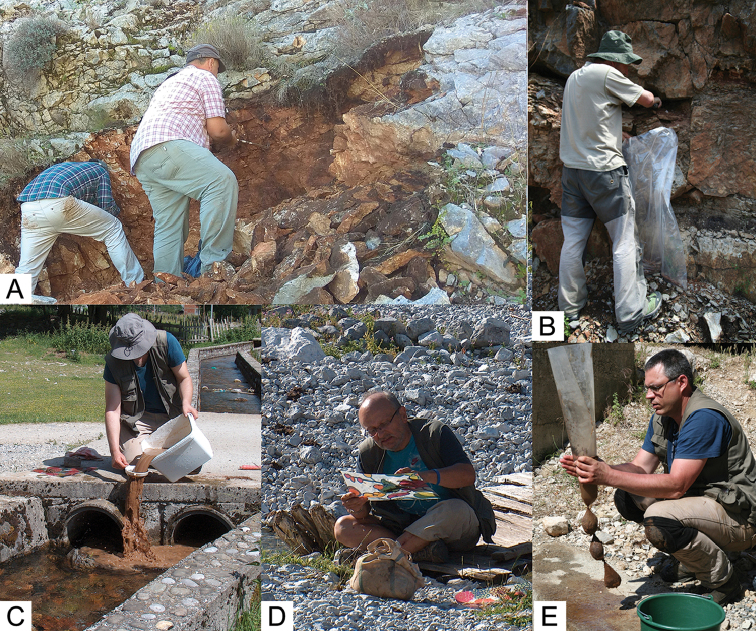
“Scratch and flotate” method to collect shells of subterranean species. **A–B** scratching out fine granulate soil material with handrakes **C** separating shells from soil particles by flotation **D** cursory visual inspection of the floated fraction **E** Storing the floated material in polyamide knee-sock.

Our sites represent a different sort of habitat type, similar to “bedrock MSS” according to the subdivision of Mammola et al. (2016), except that the rock surfaces of these sites are not covered by an evolving soil. Due to the nature of this habitat type, the use of pitfall traps or other sort of passive sampling devices was out of question. Therefore, we stayed with the “scratch and sieve” or “scratch and flotate” approach, i.e. we scratched out fine granulate material from the superficial fissures of rocks applying long and narrow hand rakes (Fig. 15A–B) and separated the shells by sieving or by flotation (Fig. 15C–E).

It is still disputed whether such a bedrock MSS habitat is characteristically different from caves or it can be considered just as the extension of the cave system, and therefore, its fauna is composed of troglobionts of wider ecological tolerance or there are certain specialized elements exclusive of MSS (see Mammola et al. 2016 for review). The role of *Spelaeodiscus* remains unclear in this respect. However, our study indicates that the bedrock MSS habitats in the Balkans harbour a large and still largely undiscovered gastropod diversity.

## Supplementary Material

XML Treatment for Spelaeodiscidae

XML Treatment for Spelaeodiscus

XML Treatment for Spelaeodiscus albanicus

XML Treatment for Spelaeodiscus albanicus albanicus

XML Treatment for Spelaeodiscus albanicus edentatus

XML Treatment for Spelaeodiscus dejongi

XML Treatment for Spelaeodiscus densecostatus

XML Treatment for Spelaeodiscus hauffeni

XML Treatment for Spelaeodiscus hunyadii

XML Treatment for Spelaeodiscus latecostatus

XML Treatment for Spelaeodiscus obodensis

XML Treatment for Spelaeodiscus unidentatus

XML Treatment for Spelaeodiscus unidentatus unidentatus

XML Treatment for Spelaeodiscus unidentatus acutus

XML Treatment for Spelaeodiscus virpazarioides
